# Diversification of *Angraecum* (Orchidaceae, Vandeae) in Madagascar: Revised Phylogeny Reveals Species Accumulation through Time Rather than Rapid Radiation

**DOI:** 10.1371/journal.pone.0163194

**Published:** 2016-09-26

**Authors:** Herinandrianina N. Andriananjamanantsoa, Shannon Engberg, Edward E. Louis, Luc Brouillet

**Affiliations:** 1 Département de sciences biologiques, Université de Montréal, Montréal, Québec, Canada; 2 Omaha’s Henry Doorly Zoo and Aquarium, Omaha, Nebraska, United States of America; Institute of Botany, CHINA

## Abstract

*Angraecum* is the largest genus of subtribe Angraecinae (Orchidaceae) with about 221 species. Madagascar is the center of the diversity for the genus with *ca*. 142 species, of which 90% are endemic. The great morphological diversity associated with species diversification in the genus on the island of Madagascar offers valuable insights for macroevolutionary studies. Phylogenies of the Angraecinae have been published but a lack of taxon and character sampling and their limited taxonomic resolution limit their uses for macroevolutionary studies. We present a new phylogeny of *Angraecum* based on chloroplast sequence data (*mat*k, *rps*16, *trn*L), nuclear ribosomal (ITS2) and 39 morphological characters from 194 Angraecinae species of which 69 were newly sampled. Using this phylogeny, we evaluated the monophyly of the sections of *Angraecum* as defined by Garay and investigated the patterns of species diversification within the genus. We used maximum parsimony and bayesian analyses to generate phylogenetic trees and dated divergence times of the phylogeny. We analyzed diversification patterns within Angraecinae and *Angraecum* with an emphasis on four floral characters (flower color, flower size, labellum position, spur length) using macroevolutionary models to evaluate which characters or character states are associated with speciation rates, and inferred ancestral states of these characters. The phylogenetic analysis showed the polyphyly of *Angraecum sensu lato* and of all *Angraecum* sections except sect. *Hadrangis*, and that morphology can be consistent with the phylogeny. It appeared that the characters (flower color, flower size, spur length) formerly used by many authors to delineate *Angraecum* groups were insufficient to do so. However, the newly described character, position of the labellum (uppermost and lowermost), was the main character delimiting clades within a monophyletic *Angraecum sensu stricto*. This character also appeared to be associated with speciation rates in *Angraecum*. The macroevolutionary model-based phylogeny failed to detect shifts in diversification that could be associated directly with morphological diversification. Diversification in *Angraecum* resulted from gradual species accumulation through time rather than from rapid radiation, a diversification pattern often encountered in tropical rain forests.

## Introduction

Madagascar is known for its rich biodiversity [1] and is a focus for evolutionary biologists who study the causes of species diversification. Many studies have been conducted using the high endemicity and diversity found in this isolated island as a model of diversification processes in various taxa [2–10]. Most of these studies came to the conclusion that species diversification resulted from rapid radiation. High morphological variation associated with low genetic divergence appeared to be a signature of the diversification processes operating on the island of Madagascar [8] as well as on many other islands such as New Zealand, Hawaii, or those of the Caribbean [11–13]. With the improvement of methods for macroevolutionary studies [14–18] it has been advocated that species diversification is not necessarily linked with adaptive radiations [19], but could be a result of a gradual accumulation of ancestral lineages through time [20]. The problems associated with studies on diversification are primarily due to insufficient data, such as incomplete taxon sampling or phylogenetic uncertainties [17,21]. Macroevolutionary studies require a good knowledge of paleontological events and eventually a fossil record to help calibrate the phylogenies, but most of the time these are lacking, making the interpretation of evolutionary histories controversial. Understanding the diversification processes of a group of organisms could be useful for biodiversity conservation [22], especially for hotspots like Madagascar [23] where priority should be given to the most valuable species due to a lack of conservation funds.

The genus *Angraecum* is the second largest group of orchids in Madagascar; it has a high morphological diversity, which makes it a good candidate for macroevolutionary studies [21]. *Angraecum* includes approximately 221 species [24,25] distributed from sub-Saharan Africa to Madagascar, the Indian Ocean Islands (IOI: Comoros, Mauritius, Reunion, Rodrigues, and Seychelles), and Sri Lanka. Madagascar is the center of diversity for the genus with *ca*. 142 species, of which 90% are endemic. The majority of species are epiphytic, but some are lithophytic. Epiphytic plants are found in the tropical rain forest across the eastern slopes of mountains or in mesic forests in the central highland, while lithophytic species are found on inselbergs or on limestone. Recent molecular phylogenetic work [24] placed *Angraecum* in subtribe Angraecinae, the latter sister to subtribe Aeridinae of tribe Vandeae. A phylogenetic reconstruction of the tribe Vandeae [26] revealed the polyphyly of Angraecinae *sensu* Dressler [27] and of many of its genera including *Angraecum* [26,28]. Accordingly, a new circumscription has been proposed by merging Angraecinae and Aerangidinae into a single subtribe Angraecinae [28], which includes 47 genera and *ca*. 762 species [24]. Even though revisions were made to resolve the polyphyly of several genera (e.g. [29,30]), similar attempts with *Angraecum* proved difficult because of limited sampling [26,28]. In general, research on *Angraecum* is complicated by the large number of species and great morphological diversity within the genus, with morphological similarities to taxa in other genera of subtribe Angraecine confusing matters further. Garay [31] proposed 19 sections to accommodate the species according to morphological descriptions (essentially floral, e.g. flower color, flower size, spur length), as was done by previous authors (e.g. [32–35]). Recent molecular phylogenic work revealed that Garay’s sections are polyphyletic [28,36]. Micheneau *et al*. [28] showed that the morphology-based classification did not reflect the molecular phylogeny, and concluded that most of the sections of Garay [31] were non-monophyletic. This study was based on Mascarene species and was lacking samples from Madagascar. Some authors proposed to remove all species that cause the polyphyly with the aim of making *Angraecum* monophyletic [36–38]. Thus, all strictly African sections were removed from *Angraecum sensu lato* [38] and elevated to generic rank (*Angraecoides*, *Dolabrifolia*, *Eichlerangraecum* and *Pectinariella*), making the taxa sampled monophyletic, but as mentioned above, the study was not comprehensive, lacking Malagasy taxa.

Although the evolution of pollinia, the adaptation to epiphytic habitat and the development of crassulacean acid metabolism (CAM) photosynthesis was shown to be involved in the earlier diversification of the Orchidaceae [39], little is known about the relationship between morphological variation and species diversification. With the morphological diversity observed in *Angraecum*, we were interested in testing whether the characters historically used to define the sections, essentially flower color, flower size and spur length, were phylogenetically useful and involved in species diversification. Questions also arose on how the diversification of the genus occurred. Here, we present a phylogenetic reconstruction of *Angraecum sensu* Garay based on molecular DNA sequence data and a more comprehensive sample of species. Using this phylogenetic framework combined with morphological data, we attempt to resolve the systematic problems existing at the sectional level, and try to understand the patterns of species diversification in *Angraecum*. Therefore, the main objectives of the current study are to (1) reconstruct the phylogeny of *Angraecum sensu* Garay [31] using a larger sample of Malagasy species and most available Angraecinae species; (2) test the monophyly of morphologically defined sections; (3) evaluate whether species diversification follows morphological diversification; (4) assess the validity of the morphological characters for taxonomy; and (5) to establish whether diversification was the result of rapid radiation or more gradual taxon accumulation. To avoid confusion, we will use *Angraecum* to designate the *sensu stricto* genus, and *Angraecum sensu lato* to identify the widest concept of the genus as defined by Garay.

## Materials and Methods

### Taxon sampling and study site

Plant tissue for DNA extraction was obtained from field collected silica gel dried samples [40] of 36% of Malagasy *Angraecum s*.*l*. and Angraecinae species. The remaining materials, in the form of sequences data, came from previous studies [26,28,36,41]. A total of 194 specimens, of which 69 were newly sampled, were included in our analyses, comprising 98 *Angraecum s*.*l*., 17 *Jumellea*, 10 *Aeranthes*, and 40 other genera (S1 Table). *Acampe ochracea*, *Aerides odorata*, *Phalaenopsis cornu*-*cervi*, *Vanda tricolor* (subtribe Aeridinae), and *Polystachya fulvilabia* (subtribe Polystachyinae) were used as outgroups based on previous phylogenetic studies [24,26,28].

The study focused on Madagascar as this is the center of diversity of *Angraecum* [42]. Sampling was carried out from 2007 to 2012 at 11 sites. The characteristics of each site are shown in Table 1 where evergreen and deciduous formations correspond to the description of Du Puy & Moat [43]. The expeditions were carried out in collaboration with Madagascar Biodiversity Partnership, the University of Antananarivo, the Ministry of Environment, Ecology and Forest, and Madagascar National Park. Research permits were issued by the Ministry of Environment, Ecology and Forest in agreement with Madagascar National Park and the University of Antananarivo. Field studies did not involve endangered or protected species.

**Table 1:** Characteristics of the sampling sites.

Sites	latitude (south)	longitude (east)	elevation (m)	forest formations
Ambohitantely	-18.19580	47.28955	1529–1639	evergreen, humid forest (low mountain)
Angavokely	-18.92172	47.73122	1569–1692	evergreen, humid forest (low mountain)
Anjozorobe	-18.39330	47.93930	1550–1612	evergreen, humid forest (low mountain)
Bemaraha	-19.04461	44.78047	45–162	deciduous, seasonally, dry (low altitude)
Lakia	-21.49475	47.89972	193–444	evergreen, humid forest (low altitude)
Mananara-Nord	-16.31261	49.78536	5–433	evergreen, humid forest (low altitude)
Manongarivo	-14.02302	48.25522	415–835	evergreen, humid forest (mid altitude)
Mantadia	-18.82641	48.44086	980–1149	evergreen, humid forest (mid altitude)
Marojejy	-32.98469	92.00141	779–865	evergreen, humid forest (mid altitude)
Torotorofotsy	-18.78208	48.43369	925–1029	evergreen, humid forest (mid altitude)
Vatovavy	-21.40777	47.94111	338–591	evergreen, humid forest (low altitude)

Because of conservation policy in Madagascar National Parks, the collection of herbarium specimens was not allowed in protected areas. Therefore, pictures were taken to serve as vouchers and were stored at the Marie-Victorin Herbarium (MT), which will be accessible in the future via Canadensys (http://data.canadensys.net/explorer/en/search). Elsewhere, voucher specimens were collected and deposited at the national herbarium of Madagascar (TAN) and MT. Overall, 32 of the 69 sampled specimens were vouchered (S1 Table).

### Morphological data

To investigate morphological evolution and diversification patterns, 39 characters (13 vegetative and 26 floral) were scored for the taxa represented in our molecular sampling. Type specimens preserved in the herbaria at Kew (K) and Paris (P), where over 80% of holotypes are located, were scored for each *Angraecum s*.*l*. species. Missing characters were documented from literature descriptions. For type specimens preserved in other herbaria (e.g. B, BM, MO) that we did not borrow and for other genera, characters were scored from photographs of living material and voucher specimens, as well as from literature descriptions [34,42,44]. Character descriptions and other background information are provided in S2 Table, while the generated matrix is presented in S3 Table. The independence of the morphometric characters was not assessed, as we did not anticipate this would unduly impact on the results.

### PCR amplification and DNA sequencing

Total DNA was extracted from 20–30 mg of silica-dried gel leaf material following the modified hexadecylmethylammonium bromide (2x CTAB, 2% (w/v)) extraction protocol of Doyle and Doyle [45]; 1% polyvinylpyrrolidone (PVP) and 0.2% of β-mercaptoethanol was added to the total volume of the extraction buffer. Three plastid DNA markers were amplified: *mat*K coding gene, *rps*16 intergeneric spacer, and *trn*L intron. The amplification of the *mat*K region was performed using the barcoding primers 472F/1248R designed by Yu *et al*. [46]. The *rps*16 region was amplified using the primers 1F/2R designed by Oxelman *et al*. [47]. The *trn*L intron was amplified using the primers 49873F/50272R designed by Taberlet *et al*. [48]. The nuclear ribosomal internal transcribed spacer ITS2 region was amplified using the primers S2F/S3R designed by Chen *et al*. [49]. The PCR reactions contained, 1X PCR reaction buffer, 2.5 mM MgCl2, 0.16 μM of each primer, 0.2 mM of each dNTP, 0.4% bovine serum albumin (BSA), 2 units of Taq DNA polymerase, 30 ng of template DNA, adjusted to a final volume of 25 μL with de-ionized water. PCR conditions are the same as described in Yu *et al*. [46]. PCR amplifications were performed on a GeneAmp PCR System 9700 thermocycler (Applied Biosystems, Foster City, CA), and resulting PCR products were purified using exonuclease I and shrimp alkaline phosphatase (ExoSAP; [50]). The purified products were cycle sequenced using a BigDye® terminator sequencing kit (Life Technologies, Carlsbad, CA). Sequences were analyzed with an Applied Biosystems 3130xl genetic analyzer at the Omaha’s Henry Doorly Zoo and Aquarium (NE, USA). Sequence fragments were aligned to generate a consensus sequence using Sequencher® 4.10 (Gene Codes Corporation; Ann Arbor, MI). Sequence data were also obtained from the Canadian Center for DNA Barcoding (part of *mat*K and ITS2) and from GenBank for previous studies [26,28,36,41]. Following automatic alignment using SeaView [51], alignments were edited manually using BioEdit [52]. All newly generated sequences have been deposited in BOLD and GenBank (S1 Table), and the matrices in TreeBase (http://purl.org/phylo/treebase/phylows/study/TB2:S19429).

### Phylogenetic analyses

Five matrices were produced: [1] combined plastid, [2] nuclear ribosomal, [3] morphological, [4] combined plastid and morphological, and [5] combined molecular (plastid and nuclear ribosomal) and morphological. They were analyzed using maximum parsimony (MP) and Bayesian analyses (BA). Tree searches under parsimony were conducted in PAUP* [53]. A preliminary heuristic search was performed with 1000 replicates of random addition sequence, tree bisection–reconnection (TBR) branch swapping, retaining twenty most parsimonious trees at each replicate. Starting with the trees kept in memory from this initial analysis, a second heuristic search was performed with TBR and 10000 trees were saved. A strict consensus tree was constructed for each analysis. Branch support was estimated using 5000 bootstraps replicates under a heuristic strategy with one random addition-sequence replicate TBR branch swapping. Bayesian analyses were performed with MrBayes [54]. The best nucleotide substitution model was selected with jModelTest2 [55] using the Akaike information criteria [56]. For all regions, the GTR+I+G model scored best and was selected. For the combined molecular and morphological matrix, data were partitioned as DNA and standard respectively. Two parallel runs of eight Metropolis Coupled Markov Chain Monte Carlo (MCMCMC) each, and four swaps per swapping cycles for 15 million generations were undertaken. Trees were sampled every 1000 generations, and the first 25% generations were discarded as burnin. The 50% majority consensus tree with Bayesian clade credibility was built from post-burnin trees.

### Estimation of divergence times

Divergence times were estimated using a relaxed molecular clock approach as implemented in BEAST [57]. The combined plastid matrix was used as input data in BEAUti. The GTR+G+I model was selected as substitution model. A relaxed lognormal molecular clock model was selected. The Yule model was selected as tree prior. The age for the root of the tree was set to a normal distribution with mean 35 million years (Ma) and a standard deviation of 3 (giving a 95% CI ranging from 30.07–39.93 Ma). Because fossil data are rare in the Orchidaceae, only three fossils having been recorded so far [58], none of them close to our group, we used the age estimate of *Phalaenopsis* [59], a member of subtribe Aeridinae sister to Angraecinae which occupies a phylogenetic position near the base of tribe Vandeae [24,60], to calibrate the stem root. The prior distribution of the ‘ucld.mean’ parameter was set to an exponential distribution (mean = 10.0, initial value = 1.0). Four separate runs were performed in BEAST with 50 million generations each, sampling parameters and trees every 1000 generations. Trees were summarized with burnin values set to the first 25% of trees sampled using TreeAnnotator and were summarized in a maximum clade credibility tree.

### Diversification analyses

In order to assess diversification patterns, we evaluated the state-dependent diversification of morphological characters using the Binary State Speciation and Extinction (BiSSE) and the MultiState Speciation and Extinction (MuSSE) models implemented in the R package ‘diversitree’ [61], and the speciation/extinction and phenotypic/evolution models using BAMM [62]. Four floral characters were chosen for these analyses because of their taxonomic interest: flower colors, flower size, spur length, and labellum position (S2 Table). Three of these characters (flower color, flower size and spur length) have been used previously to delineate sections in *Angraecum* (e.g. [31,34]), while the labellum position was added because it appeared useful for sectional delimitation. Since these methods require ultrametric and fully bifurcating trees, we used the BEAST maximum clade credibility tree as input.

Two characters were analyzed using BiSSE: flower color and labellum position. Since we were interested in the effect the green and white flower colors may have on diversification, the taxa that did not fit in these two colors (for instance, pink or purple flowered taxa) were excluded from the analyses using the ‘drop.tip2’ function of the R package ‘phyloch’ [63]. Four models were tested: (M1) a full model that allows all parameters' (***λ*:** speciation rate, ***μ***: extinction rate, ***q*:** transition rate) to vary, (M2) a constrained model allowing speciation and transition rates to vary while keeping extinction rates equal between states (***μ***0 ~ ***μ***1), (M3) a constrained model allowing extinction and transition rates to vary while keeping speciation rates equal between states (***λ***0 ~ ***λ***1), and (M4) a constrained model allowing speciation and extinction rates to vary while keeping transition rates equal between states (***q***0 ~ ***q***1). Two characters were analyzed with MuSSE: flower size and spur length. To categorise these continuous characters, partition analyses using K-means were conducted (S1 Fig and S4 Table) as implemented in the R package ‘stats’ [64,65]. Both characters were treated as ordered. A preliminary run was performed to determine the best transition model. Only transitions that we considered biologically plausible were tested (e.g.: transitions between very small flower to very large flower, or between very short spur to very long spur are less probable). For flower size, which has four character states (1: small, 2: medium, 3: large, 4: very large), three models were tested: (M1) a full model that allows all parameters (***λ***, ***μ***, ***q***) to vary, (M2) a constrained model where character evolution was possible in both directions between neighboring states (1 ↔ 2 ↔ 3 ↔ 4), and (M3) a constrained model where evolution could be either unidirectional or bidirectional between neighboring states (1 ←2 ←3 ↔ 4). For spur length, which has four character states (1: short, 2: medium, 3: long, 4: very long), three models were also tested: (M1) a full model that allows all parameters (***λ*,** ***μ***, ***q***) to vary, (M2) a constrained model where character evolution was bidirectional and possible only among neighboring states (1 ↔ 2 ↔ 3 ↔ 4), and (M3) a constrained model where character evolution could be either unidirectional or bidirectional between states that are not necessarily adjacent (reticulated) (1 ←2 ↔ 3 ↔ 4, 1←3, 1←4). Having established the best fit transition model, we used it as a constraint while testing the three models of diversification: (M1) an unconstrained model that allowed all parameters (***λ***, ***μ***, ***q***) to vary, (M2) a constrained model allowing speciation and transition rates to vary while keeping extinction rates equal between states, and (M3) a constrained model allowing extinction and transition rates to vary while keeping speciation rates equal between states. After testing the models with BiSSE and MuSSE, the posterior probabilities of the parameters were computed under a Bayesian framework after setting the priors to be exponential. The MCMC was run for 10000 generations sampling parameters every 100 generations, and the posterior distributions of parameters were summarized using the function ‘profiles.plot’ implemented in ‘diversitree’ [61].

Many criticisms have been made concerning the SSE family (BiSSE, MuSSE, GeoSSE, etc.) type I error rate and limitations [66,67]. It has been reported that “within-clade pseudoreplications” might result in erroneously significant results [66]. The use of additional methods like BAMM has been proposed to reinforce the results implemented under SSE’s [67]. Both methods use different approaches but are complementary: MuSSE looks at the effects of character states on diversification, while BAMM quantifies diversification rates and detects macroevolutionary rate shifts events (speciation, extinction and phenotypic evolution) across phylogenetic trees.

For the speciation/extinction model implemented in BAMM, we estimated our total sampling at 40% and we fractioned the data according to the number of species per genus being represented in our phylogeny (S4 Table) in order to reduce the bias in estimating parameters under an assumption of incomplete sampling [62]. To reduce the weight of the outgroup taxa we excluded them from the analyses using the ‘drop.tip2’ function of the R package ‘phyloch’ [63]. For the phenotypic/evolution model, we assessed the regime of morphological evolution of two of the four characters mentioned above, flower size and spur length (S4 Table), since this model only treats continuous characters. For each model (speciation/extinction and phenotypic/evolution), BAMM was run for 5,000,000 generations and parameters were sampled every 1000 generations. The parameter priors were set using parameters generated from the R package ‘BAMMtools’ [18]. The rate shift configurations and the rate through-time generated from ‘bammdata outputs’ were analyzed using the R package ‘BAMMtools’.

### Character state reconstruction

We examined the character evolution of four floral characters previously used in taxonomy (flower colors, flower size, spur length, and labellum position) using the Markov discrete character evolution [14] as implemented in the R package ‘diversitree’ [61]. Because this method requires ultrametric and fully bifurcating trees, we used the BEAST tree as input. No constraint was applied to the analyses leaving all parameters free.

## Results

### Phylogenetic relationships

Of the 69 newly sampled specimens that we sequenced, all were fully amplified with *mat*K for a total length of 942 base pairs (bp); 66 with *rps*16 (1192 bp) except *Angraecum sterophyllum*, *A*. *rhynchoglossum* and *Lemurella papillosa* which failed to amplify; and 65 with *trn*L (1553 bp) except *A*. *pseudofilicornu*, *A*. *rhynchoglossum*, *Oeoniella polystachys* and *Oeonia rosea*. Only 13 of 69 samples were sequenced with ITS2 for a total length of 381 bp. When combined with GenBank sequences data, each individual matrix was composed of 190 taxa for *mat*K, 144 for *rps*16, 170 for *trn*L and 88 for ITS2. Because of the small amount of samples available with ITS2, we decided to include only taxa present in this matrix to run the combined molecular (plastid and nuclear) and morphological analyses for a total of 88 taxa and 4107 characters. Therefore, the combined plastid matrix contained 3687 characters and 194 taxa. Sequences that were unavailable were treated as missing data. For the *trn*L intron, the ambiguous variable sites (360 bp) were excluded from our analyses. For the whole data set, 2361 characters were constant, 467 (13.8%) variable characters were parsimony-uninformative, and 537 (16%) characters were parsimony-informative. The combined plastid matrix produced five trees of 2202 steps with a consistency index (CI) of 0.56 and a retention index (RI) of 0.77.

**Fig 1:**
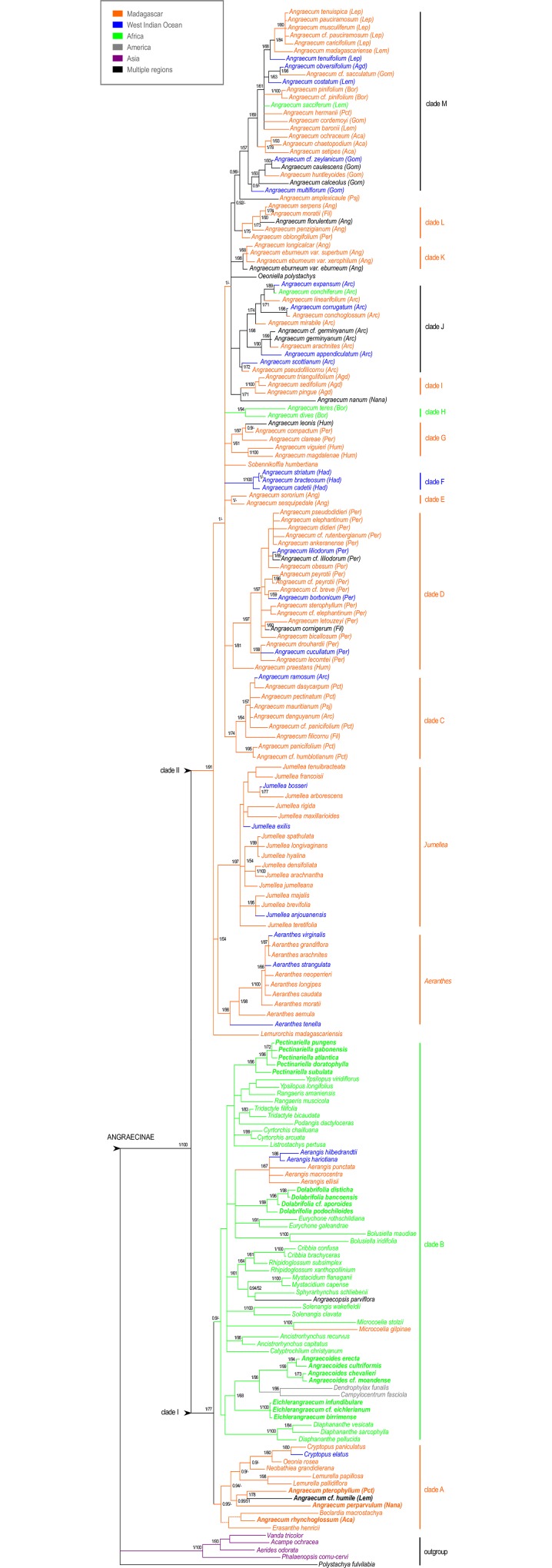
Phylogenetic relationships within subtribe Angraecinae. 50% Bayesian majority-rule consensus tree from combined plastid data (*mat*K, *rps*16 and *trn*L). Values above branches or at nodes represent posterior probability (PP) and bootstrap percentage (BP) support. Dashes represent branches that collapsed in MP strict consensus tree. Colors represent geographic distribution of taxa; in bold are *Angraecum sensu* Garay species. Abbreviations in brackets denote sections *sensu* Garay: Aca = *Acaulia*, Agd = *Angraecoides*, Ang = *Angraecum*, Arc = *Arachnangraecum*, Bor = *Boryangraecum*, Chl = *Chlorangraecum*, Fil = *Filangis*, Gom = *Gomphocentrum*, Had = *Hadrangis*, Hum = *Humblotiangraecum*, Lem = *Lemurangis*, Lep = *Lepervenchea*, Pct = *Pectinaria*, Per = *Perrierangraecum*, Psj = *Pseudojumellea*.

The MrBayes, BEAST, and MP analyses were congruent. The 50% majority-rule consensus tree from Bayesian analyses of the combined plastid matrix is displayed in Fig 1. Our results support a monophyletic subtribe Angraecinae (PP 1.0, BP 100). Two well-supported clades were identified within Angraecinae: clade I (PP 1.0, BP 77) comprised of Malagasy, IOI, African and American genera, and clade II (PP 1.0, BP 91) with Malagasy and IOI genera. Clade II had more branch support and showed more resolution than clade I. Two main subclades are observed within clade I: a Malagasy-IOI clade A (PP 1.0), and an African-American clade B (PP 1.0). Four Malagasy *Angraecum s*.*l*. species (*A*. *perparvulum*, *A*. cf. *humile*, *A*. *pterophyllum*, and *A*. *rhynchoglossum*) are nested within clade A. Three major subclades were observed in clade II: *Aeranthes* (PP 1.0, BP 98), *Jumellea* (PP 1.0, BP 97), and *Angraecum* (PP 1.0). Here, we define *Angraecum* as a monophyletic group including all *Angraecum* species and all other taxa nested within it in clade II. Based on branch support (PP, BP) and morphological resemblance, eleven clades are observed within *Angraecum* (Fig 1, clade C to M). From the base to the top of the tree are: clade C (PP 1.0, BP 74) comprised members of sections *Pectinaria*, *Pseudojumellea*, *Arachnangraecum* and *Filangis*; clade D (PP 1.0, BP 81) comprising member of sections *Perrierangraecum*, *Angraecum*, *Arachnangraecum* and *Filangis*; clade E (PP 1.0) which includes *A*. *sesquipedale*, and *A*. *sororium* of section *Angraecum*; clade F (PP 1.0, BP 100) section *Hadrangis*; clade G (PP 1.0, BP 61) with section *Humblotiangraecum* and a member of section *Perrierangraecum*; clade H (PP 1.0, BP 94) section *Boryangraecum*; clade I (PP 1.0, BP 100) section *Angraecoides*; clade J section *Arachnangraecum*; clade K (PP 1.0, BP 98) section *Angraecum* (*A*. *eburneum*); clade L (PP 1.0, BP 75) comprised of members of sections *Angraecum* and *Pseudojumellea*; clade M (PP 1.0, BP 57) composed of sections *Acaulia*, *Boryangraecum*, *Chlorangraecum*, *Gomphocentrum*, *Lemurangis*, and *Lepervenchea*. Clades E and J received weak support despite the fact that their positions were supported in a strict consensus tree. Two species are not included in any of the clades, *A*. *nanum* sister to clade I (PP 1.0, BP 72), and *A*. *amplexicaule* intermediate between clade L and M (Fig 1). Furthermore, two Malagasy genera, *Oeoniella* and *Sobennikoffia*, are nested within *Angraecum*. Even though many clades were strongly supported within *Angraecum*, resolution between clades was low.

The phylogeny obtained from ITS2 lacked resolution and was slightly incongruent with the combined plastid phylogeny (S2 Fig). Notably, the Malagasy Angraecinae genera *Aeranthes*, *Jumellea*, and *Lemurorchis* were embedded within *Angraecum*, rendering it paraphyletic. Nonetheless, all represented clades within *Angraecum* appeared to be congruent with those in the plastid data (Fig 1). Our combined plastid and morphological analyses yielded a topology that is congruent with the combined plastid analyses with slight differences in branch supports (S3 Fig). Within *Angraecum*, clade M (PP 1.0, BP 75) gained support while clade D became weaker. The position of *Lemurorchis* was ambiguous, forming a polytomy with the *Aeranthes*–*Jumellea* clade in the molecular analyses while it was embedded with *Angraecum* in the combined analyses. The morphological analyses alone supported the monophyly of Angraecinae *sensu* Carlsward *et al*. [26], with many polytomies observed within the clade (result not shown). According to the morphological phylogeny, most genera were monophyletic (*Aeranthes*, *Jumellea*, and most of the African genera) except *Angraecum*, where conflicts were also observed when compared to the plastid tree. The combined molecular (plastid and nuclear) and morphological analyses yielded a result that is incongruent with the combined plastid phylogeny, but similar to the nuclear topology (result not shown).

### Divergence time estimates

The maximum credibility tree of the calibrated relaxed molecular clock analysis of Angraecinae is shown in S4 Fig. Our results suggest that Angraecinae shared a most recent common ancestor (MRCA) in the late Oligocene (~ 26.1 Ma, giving a node height highest posterior density (HPD) intervals at 95% ranging from 18.2–33.5 Ma), and started to diverge in the early Miocene (S5 Table). Diversification started at approximately 21 Ma (95% HPD: 14.6–27.8 Ma) for clade I and 17.1 Ma (95% HPD: 11.7–23.3 Ma) for clade II. In clade I, the African-American clade B diverged at approximately 19.46 Ma (95% HPD: 13.7–25.9 Ma), while the Malagasy-IOI clade A diverged at approximately 18.1 Ma (95% HPD: 12.1–24.5 Ma). Within clade II, *Angraecum*, *Aeranthes* and *Jumellea* started to diversify at around 15.5 Ma (95% HPD: 10.4–20.6 Ma), 10.3 Ma (95% HPD: 5.8–15.1 Ma), and 7 Ma (95% HPD: 4.7–11 Ma) respectively. By counting the number of taxa (nodes) on our calibrated maximum credibility tree (S4 Fig), it appears that 60% of the speciation events within the orchid genera *Aeranthes*, *Angraecum* and *Jumellea* happened during the Pliocene-Pleistocene. *Angraecum* section *Hadrangis* which is endemic to the Mascarene Islands diverged at approximately 1.66 Ma (95% HPD: 0.4–3.3 Ma), and the divergence time for the two species *A*. *bracteosum* and *A*. *striatum* that are endemic to Reunion is estimated at 0.2 Ma (95% HPD: 0–0.7 Ma).

### Species diversification

**Fig 2:**
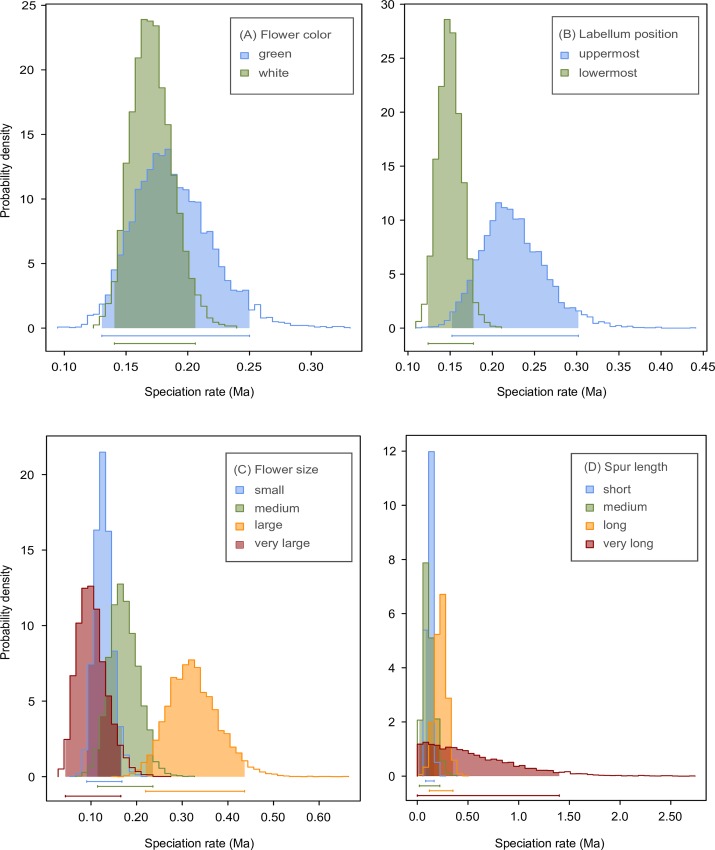
**Posterior probability distributions for the speciation rates (in Ma) of morphological characters using equal rate speciation (*μ*0 ~ *μ*1) with the BiSSE model and equal rate extinction (*λ*i ~ *λ*j) with the MuSSE model**: flower color (A), labellum position (B), flower size (C), and spur length (D). Abbreviation: v, very.

Results from BiSSE showed that the second model (***μ***0 ~ ***μ***1) received the best AIC score for flower color and labellum position (S6 Table). The green and white colors had similar rates of speciation (Fig 2A), while the uppermost labellum showed a higher speciation rate compared to the lowermost one (Fig 2B). Results from MuSSE showed that the third transition model (1 ←2 ←3 ↔ 4) had the best AIC score for flower size, while the full model received the best score for spur length (S6 Table); these models were used to test diversification. The diversification model with extinction rates equal between states received the best score for flower size and spur length, suggesting that character states have an effect on speciation. Large flowers showed higher speciation rates compared to small, medium and very large flowers (Fig 2C). All spur length states had a similar effect on speciation rates (Fig 2D).

**Fig 3:**
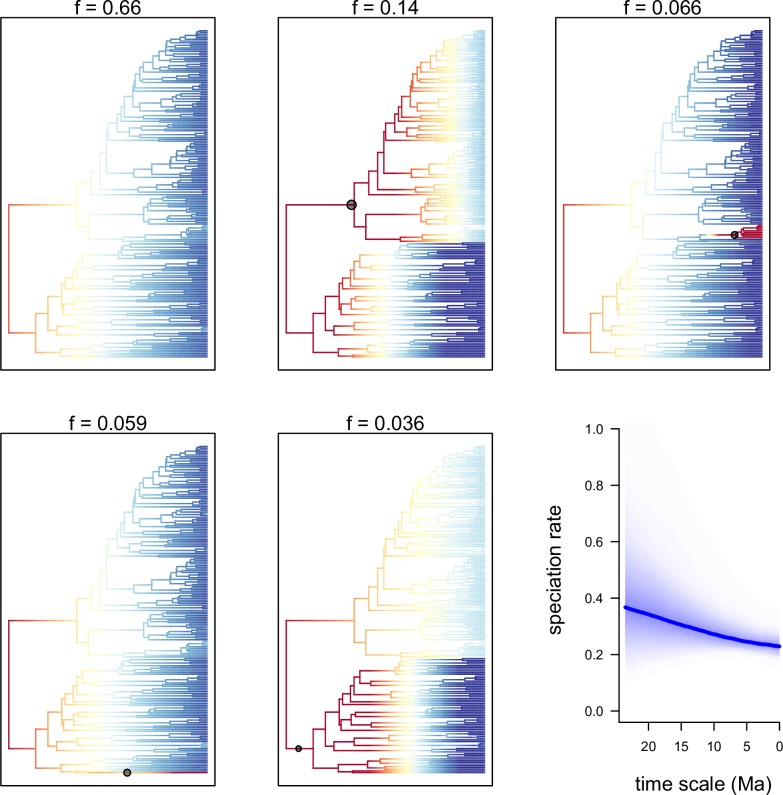
Configuration shifts from the 95% credible set sampled by BAMM from the Angraecinae phylogeny and evolutionary rates through time. The intensity of colors on branches reflects the relative probability density of speciation rates (cool colors = slow, warm = fast). Black circles denote the position of the macroevolutionary regime shifts present in each sample. Blue curve indicates the mean speciation rate-through-time trajectory of Angraecinae in million years. Values above dendograms (f: configuration sampled frequency) indicate the marginal probability of rate shifts observed on branches across the posterior distribution of macroevolutionary rate shift configurations.

The speciation/extinction model from BAMM revealed 5 distinct configuration shifts from the 95% credible set (Fig 3, percentages indicated at top of trees) of which 66% of the samples in the posterior distribution showed no shift, 14% showed a single shift at the node of clade II, 6.6% had one within the *Aeranthes* clade, 5.9% had one shift at the branch of *Beclardia macrostachya*, and 3.6% of the posterior distribution had one shift at the branch of clade I. Our BAMM results showed a gradual decline of speciation rate-through-time (RTT) for Angraecinae, starting from approximately 0.39 Ma during the Miocene to 0.23 Ma towards the present (Fig 3).

**Fig 4:**
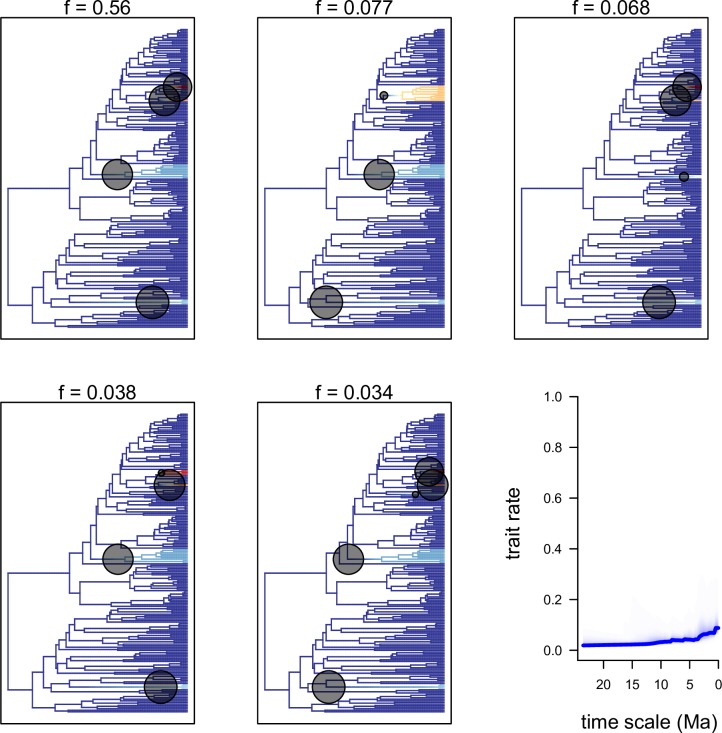
Best configuration shifts from the 95% credible set sampled by BAMM for the evolution of spur length across the phylogeny of Angraecinae. Color intensity on branches reflects the relative probability density of the instantaneous rate of phenotypic evolution. Black circles denote the position of the macroevolutionary regime shifts present in each sample. Blue curve denotes the mean evolution rate-through-time trajectory.

The phenotypic/evolutionary model showed 32 distinct configurations on spur length. The five that received the best sample frequencies are displayed in Fig 4. Four main shifts are observed, one at the branch of the *Eichlerangraecum* clade, one at the branch of clade A, one at the branch of *Angraecum appendiculatum*, and one at the branch of *Angraecum corrugatum*. The RTT phenotypic evolution showed an increased rate in spur length starting from 0.02 Ma during the Pliocene to 0.08 Ma in the Pleistocene and to the present (Fig 4). No shift has been detected regarding flower size within Angraecinae and the RTT phenotypic evolution was constant (S5 Fig).

### Ancestral state reconstructions

**Fig 5:**
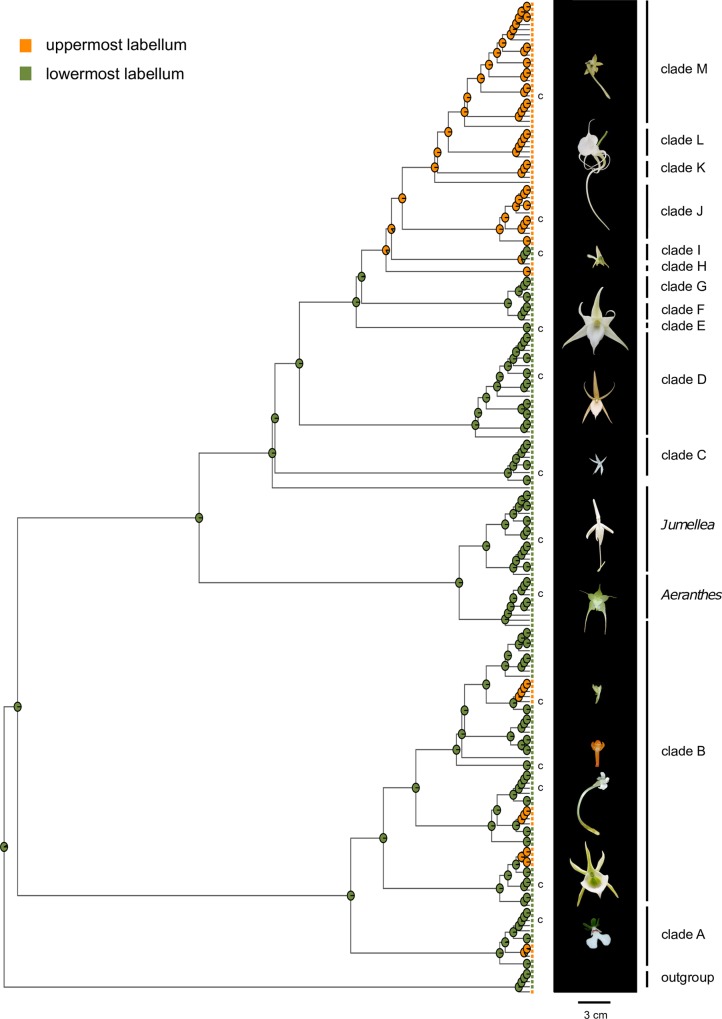
**Ancestral state reconstructions of floral characters in Angraecinae implemented in ‘diversitree’**; colors represent character states of the labellum position (uppermost and lowermost) and pie charts represent the probability of ancestral states at nodes. C denotes taxa illustrated in the pictures to the right to represent the flower shape of each clade except for *Jumellea* which is represented by *Jumellea comorensis* (not sampled in the phylogeny). Photo: Andriananjamanantsoa.

Our results showed that a labellum in the lower position is plesiomorphic in Angraecinae, while an upper labellum is apomorphic and evolved at least five times independently (Fig 5). White flowers appear to be symplesiomorphic in Angraecinae, while green flowers are apomorphic and evolved independently several times in Angraecinae and twice in *Angraecum* (S6A Fig). A medium flower size appears to be the ancestral state in Angraecinae, while large and small flowers are derived (S6B Fig). Long spur is the ancestral state and short spur is derived and arose several times independently (S6C Fig). Our results showed that the color (green), the size (small and large), and the spur length (short) of flowers are homoplasic within *Angraecum*. The taxa that showed uppermost labella appeared to be monophyletic and received very strong support in the phylogeny (*Angraecoides*, *Dolabrifolia*, *Pectinariella*, and *Angraecum* clades H to M).

## Discussion

### Systematics of Angraecinae

Our results confirmed the monophyly of the Angraecinae and the distinctiveness of the Malagasy–IOI clades from the African–American clade [26,28]. Our results also demonstrated the polyphyly of *Angraecum s*.*l*. Indeed, in addition to the African sections already transferred to new genera [38], four Malagasy species were embedded within clade A: *Angraecum* cf. *humile*, *A*. *perparvulum*, *A*. *pterophyllum*, and *A*. *rhynchoglossum*, belonging to sections *Lemurangis*, *Nana*, *Pectinaria*, and *Acaulia*, respectively [31]. Micheneau *et al*. [28] first reported the existence of a rare *Angraecum* species from Madagascar and the Mascarenes (*A*. *sp*. TP84, section *Nana*) in clade A. In our treatment, this taxon proved to be unrelated to ours and embedded within *Lemurella* (result not shown). To keep *Angraecum* monophyletic, these species must be removed from the genus.

The problem we encountered while amplifying the ITS region was primarily caused by endophytes: instead of amplifying orchid DNA, the primers amplified fungi. This was also reported by several authors working on Orchidaceae (e.g. [26]). The incongruence observed with the morphological phylogeny illustrates the existence of homoplasies in Angraecinae and the difficulty of delineating natural groups using morphology alone in this group. The variation in branch support between the combined plastid and combined plastid + morphological analyses are probably due to synapomorphic characters that were observed in some clades but were lacking in others, as well as the numerous plesiomorphic characters that are encountered in subtribe Angraecinae. The poor resolutions observed within or between clades were probably due in part to a lack of sampling or caused by a low number of markers at the positions analysed. More regions are needed to increase the resolution of the phylogeny, especially the relationships between clades.

### Systematics of *Angraecum*

The paraphyly observed in the ITS2 data may be due to a lack of sampling and to low genetic variation. Carlsward *et al*. [26] had detected paralogy in the ITS region in the Malagasy Angraecinae and most notably in clade II and therefore excluded this region from subsequent analyses, including the genera *Aeranthes*, *Jumellea*, *Lemurorchis*, *Oeoniella* and *Sobennikoffia*. Since most of the sequences we used to reconstruct the ITS phylogeny came from their work, except for *Angraecum* which came mostly from our samples, and given that only ITS2 was sequenced by us, we believe that it is difficult to reconcile the data at this point. Given the potential variability of ITS sequences, it might be worth attempting to resolve the problems of paralogy and primer.

The plastid results also showed that *Angraecum* is paraphyletic. Two Malagasy genera, *Oeoniella* and *Sobennikoffia*, are nested within this clade, as was reported by Carlsward *et al*. [26]. However, *Aeranthes* and *Jumellea* form natural groups sister to *Angraecum* as also reported by Micheneau *et al*. [28]. Schlechter [33] admitted the close resemblance between *Oeoniella* and *Angraecum*, but emphasized the differences in column shape and stipe length. These two genera differ from *Angraecum* in their labellum shape. If we look closer at *Oeoniella* and unfold the labellum, the flower looks similar to *Angraecum eburneum*, except that the perianth is soft, the labellum spurless, and the ovary untwisted. *Sobennikoffia*, a small genus of four species, was previously included within *Angraecum* [25] until transferred to a new genus by Schlechter [32] because of the three-lobed labellum.

Of the 16 sections of Garay [31] represented in our phylogeny, only one is monophyletic, the Mascarene section *Hadrangis*; all other sections are paraphyletic (*Gomphocentrum*, *Lemurangis* and *Lepervenchea*) or polyphyletic (*Acaulia*, *Angraecum*, *Angraecoides*, *Arachnangraecum*, *Boryangraecum*, *Filangis*, *Humblotiangraecum*, *Pectinaria*, *Perrierangraecum* and *Pseudojumellea*). Micheneau *et al*. [28] mentioned the unnaturalness of Garay’s sections and pointed out the complexity of dealing with the sections with small greenish flowers. We decided to not consider *A*. *nanum* within clade I because of their morphological differences. Although these species produce green flowers, *A*. *nanum*, the sole member of section *Nana*, is characterized by tiny plant with racemose inflorescences and minute flowers, while clade I is characterized by erect or pendent plants with one-flowered inflorescences and larger flowers. The position of *A*. *amplexicaule* is ambiguous but in the BEAST analyses it was embedded within clade L (S4 Fig). Morphologically, *A*. *amplexicaule* has an inflorescence and flower shape similar to those of species in clade L, except that in clade L, the habit is more robust and the leaves are coriaceous. Despite the weak support, we decided to include *A*. *pseudofilicornu* and *A*. *scottianum* in clade J because all species in this clade share the same inflorescence type and flower shape except that the sepals and petals are reduced and the habit crassulescent for the two species. Furthermore, in the ITS2 topology *A*. *scottianum* was embedded within the clade (S2 Fig).

The morphological characters (type of inflorescence, flower size and color, and spur length) used by many authors (e.g. [31,32,34,42,44]) to delineate sections in *Angraecum* are of limited taxonomic interest and their positions on the phylogeny are not coherent. Spur length and flower size are generally correlated (small flowers have short spur and large flowers have long ones), with the exception of *A*. *appendiculatum* and *A*. *corrugatum*. These two Mascarene species, often considered peloric forms of *A*. *arachnites* and *A*. *conchoglossum*, respectively [28,31,68], instead appear to have lost or changed the genes responsible for labellum and spur development [69]. The phenotypic plasticity observed in these two species was considered to be the result of species radiation [28], but colonization of new habitats (with new selection regimes) might affect gene expression that is responsible for floral development [70–74]. Chang *et al*. [72] showed that the size and shape of sepal/petal/labellum in *Oncidium* were regulated by the OMADS5 gene. These *Angraecum* species are spurless, the labellum was changed from suborbicular concave, typical in section *Arachnangraecum*, to linear-lanceolate, and they became self-pollinated [75]. There is no evidence here of a loss of bilateral symmetry (there is still a slight zygomorphy) that would be expected in peloric flowers. Alternately, variation in spur length could be due to genetic drift [76,77]. For instance, Stewart *et al*. [42] observed that spur length in subspecies of *A*. *eburneum* was shorter the further away from Madagascar a subspecies was. One of the possible causes of flower similarity could be convergent evolution as observed in clade C and the two African genera *Dolabrifolia* and *Pectinariella* previously placed in *Angraecum* section *Pectinaria* [31], or in section *Humblotiangraecum* (clade G) and the African genus *Eichlerangraecum* (Fig 1).

### Labellum position: a missing character to delineate sections in *Angraecum*

After evaluating several morphological characters (S2 Table), we noted that the position of the labellum, uppermost or lowermost, was a good indicator of clade boundaries in *Angraecum* (Fig 5). The degree of resupination in an orchid flower can vary from 0° to approximately 360° depending on the inflorescence [78], and may be specific to a taxon. The 180° resupination results in the lowermost labellum position and a 360° resupination (double twist of pedicel or ovary) in the uppermost labellum position. The position of the labellum has been used in Orchidaceae to delimit sections within genus *Bulbophyllum* [79], and in Gesneriaceae to delimit the genus *Alloplectus* [80]. The newly defined African genera [38] *Angraecoides*, *Dolabrifolia*, and *Pectinariella* are also characterized by an uppermost labellum. Our results showed that Clades C to G have lowermost labella, while clades H to M are composed exclusively of species with uppermost labella. The dispersion of the species of section *Angraecum sensu* Garay (Fig 1: clade E vs clade K) in the phylogeny is a concrete example demonstrating the usefulness of this character to delimit sections. The labellum position is difficult to observe on herbarium sheets and often the only way to clearly see it is on living specimens. This could explain the fact that none of the original species descriptions mentioned this character [31,32,34]. Given these new findings, species descriptions and generic classifications should be updated.

### Temporal framework and paleoclimate events in *Angraecum*

Our age estimate for Angraecinae and *Angraecum* is approximately 4 Ma older than that obtained by Micheneau *et al*. [81]; the age of two endemic species of the Reunion Island (*A*. *bracteosum* and *A*. *striatum*) are younger in our results, however. This can be explained by our calibration. We set the root of the tree to 35 ± 4.9 Ma following Gustafsson *et al*. [59], while Micheneau *et al*. [81] followed Ramirez *et al*. [82] and fixed it at 30.37 ± 3.44 Ma. The main difference between the two calibrations is based on the number of fossils used in the analyses: Ramirez *et al*. [82] used a single fossil, whereas Gustafsson *et al*. [59] included three to calibrate their phylogeny of the Orchidaceae. Furthermore, Micheneau *et al*. [81] used island ages as constraints in their analysis. Since no fossil was available to directly calibrate our phylogeny, these age estimates remain approximations [83]. Nonetheless, our calibration is consistent with the divergence time of the Vandeae estimated by Givnish *et al*. [39], and our divergence time estimate for *Angraecum* corresponds well with the diversification age of most Malagasy Angiosperm endemic genera [84].

During the Miocene, the climate of Madagascar shifted gradually from cool dry to warm humid. Contraction of the arid forest and expansion of the current tropical forest in Madagascar have been documented as a result of the northern migration of the island towards the equator [84,85]. This migration, together with the establishment of the trade winds, and later the monsoons [85,86], increased moisture levels throughout eastern Madagascar, thus promoting diversification of epiphytes like *Angraecum* and the most diverse orchid genus *Bulbophyllum* [79]. The high number of speciation events observed during the Pliocene-Pleistocene [5.333 Ma to 0.011 Ma] could be explained by glaciation events, where the climate fluctuated repeatedly from cold to warm [87], or the increase in the monsoon systems’ activity during the late Miocene [86]. This increase in orchid taxa which coincides with the radiation bursts observed in Malagasy tree ferns during the same period [3] may reflect the presence of humid and warm climate promoting diversification. Moreover, species richness in some Malagasy vertebrate groups were also reported to be influenced by clade age and of particular relevance, adaptability to rainforest habitats [88]. However, the 60% increase in speciation events observed within the orchid genera *Aeranthes*, *Angraecum* and *Jumellea* during the Pliocene-Pleistocene is difficult to reconcile with the smooth steady decreases in species diversity depicted by the BAMM analysis (Fig 3), and require further research to resolve.

### Diversification in *Angraecum*

The BiSSE and MuSSE results revealed that flower size and labellum position appear associated with the diversification of Angraecinae and *Angraecum*. An uppermost labellum concurred with a higher speciation rate compared to a lowermost labellum (Fig 2B). Medium and large flowers were associated with higher speciation rates when compared to minute, small or very large flowers (Fig 2C). The overlap in the posterior probabilities observed in flower color and spur length (Fig 2A and 2D) could be interpreted as an equal rate of speciation between color and length categories. Floral divergence in Orchidaceae has been associated with pollinator shifts [89]. Fischer *et al*. [79] pointed out the importance of flower orientation in plant evolution and on species diversification. Lowermost labella serve as landing platforms for pollinators [79], while uppermost labella are associated with either autogamy or a switch to pollinating insects that prefer walking rather than flying [90]. Long spurs have been shown to be associated with specific pollinators in *Angraecum* [91,92]. Because of this specificity, long-spurred flowers are more efficiently pollinated [93–96], though it does not necessarily result in a greater speciation rate, as appears to be the case in *Angraecum*.

The speciation/extinction analyses obtained from the BAMM model could be resumed to three best configuration shifts in Angraecinae, one shift at the MRCA of clade I, one at the MRCA of clade II, and one within the *Aeranthes* clade (Fig 3). BAMM detected general shifts where diversification could potentially have arisen (at the MRCA of *Aeranthes*, *Angraecum*, and *Jumellea*), but could not detect evidence in specific clades associated with diversification regimes that we expected within *Angraecum* (the shift from lowermost to uppermost labella (Fig 2B), or the high speciation rate detected by BiSSE with flower color (Fig 2A). A lack of performance of the statistical models used in BAMM was pointed out by Rabosky and Goldberg [67]. The shift detected within *Aeranthes* could be a bias of incomplete sampling (Fig 3). Within *Jumellea* we selectively sampled at least one representative of each section of the genus [41], while sampling was more random in *Aeranthes*. Better samples are required before any conclusion on the diversification regimes operating in this clade can be drawn.

The phenotypic/evolution analyses obtained from BAMM model showed that there was no shift associated with labellum size, while at least four shifts were detected with spur length (Fig 4). This leads us to conclude that flower size is evolutionary constant within clades in the whole subtribe Angraecinae, while the evolution of spur length is more variable in some clades than in others. Rakotoarivelo *et al*. [41] pointed out the lability of spur length in *Jumellea*, and its inefficiency on delimiting sections. Since flower size is generally conserved within clades, it is difficult to ascertain the extent to which this character may influence speciation, even though this character usually is associated with pollinator type. The shifts observed in clade C and J with spur length evolution suggest that these clades are the most phenotypically diverse in the whole Angraecinae. However, it does not necessary mean that species diversification is associated with the rate of spur length evolution. If we look at clade C, there is no phylogenetic signal showing that short or long spurs led to more speciation, which excludes all hypotheses of high rates of diversification associated with this character. The rate shifts observed in clade J were caused by the two Mascarene species *A*. *appendiculatum*, and *A*. *corrugatum*.

Our BAMM analyses showed a slowdown in time of the diversification rate in Angraecinae in general (Fig 3). To test for homogeneity in the diversification rate through time, we calculated the gamma statistic of Pybus and Harvey [15] as implemented in the R package ‘ape’. Our significantly negative result (**γ** = – 2.204816) rejected the null hypothesis for a constant-rate, suggesting that the speciation rate was initially higher but slowed gradually through time, which is congruent with the BAMM results. This early burst in lineages could be explained by the fact that *Angraecum* species developed a crassulacean acid metabolism (CAM) that allows them to tolerate desiccation [97]. Whitman *et al*. [98] reported that lithophytic Angraecinae are tolerant to limited moisture availability, and chronic bushfire did not kill the population of *Angraecum sororium* and *Jumellea rigida* but only reduced their expansion. But diversification slowdown could be also the result of ecological limitation, competition, access to pollinators, species carrying capacity [99,100], or maintenance of niche similarity [101]. Lineage diversification is usually high right after colonization of a new niche, but slows through time as niches get occupied and ecological conditions for speciation decrease [8]. Scantlebury *et al*. [22] reported that diversification slowdown in Malagasy fauna is a general pattern of adaptive radiation like that observed in amphibians and birds [5,8]. However, it has been shown that the diversification pattern of the tropical rain forest has been an accumulation of lineages through time and not sudden adaptive radiations [20]. Our results appear to support this hypothesis.

It is intriguing that each clade in *Angraecum* is associated with specific floral characters. *Aeranthes* and *Jumellea* are species rich and occupy the same ecological niche as *Angraecum*. The three genera diverged approximately at the same time, but *Angraecum* has more species. If we look at the morphological differentiation between the three genera, *Jumellea* and *Aeranthes* have distinctive characters that characterize them as clades, while *Angraecum* has variable characters specific to each subclade. The diversity of *Angraecum* could perhaps be partly explained by its morphological evolution. Taxonomic diversity has been demonstrated to be complemented by morphological and ecological diversity [102]. Pollinators could have played a significant role in this diversification process. Little is known about *Angraecum* pollinators, but we know now that it is pollinated by different kinds of pollinators [75,103–105], not only hawk-moths as originally thought [91,92,106]. Micheneau *et al*. [28] reported that the radiation of *Angraecum* in the Mascarenes was caused by a change in pollinators on these islands. The absence of the original pollinators is considered to have resulted in auto-pollination in some Angraecinae species [75,107], Hermans and Hermans [108], which could be associated with a decrease or loss of rewards such as fragrance or nectar in the species [75].

## Conclusion

The present study presents the most comprehensive phylogenetic reconstruction of genus *Angraecum* to date including all the sections *sensu* Garay except the African section *Afrangraecum*. With the African sections removed [38] *Angraecum* currently includes *ca*. 190 taxa (including varieties). Our results confirmed the paraphyly and polyphyly of *Angraecum* sections, but showed certain features of morphology to be consistent with phylogeny. Principally, the position of the labellum, lowermost or uppermost, allowed us to delineate several clades. An updated systematic revision of the genus is required considering these findings. Our study revealed that many characters are associated with species diversification of *Angraecum*, the orientation of the labellum being one. However, our analyses failed to detect shifts that could have been caused by morphological diversification. Overall, the evolution and diversification of *Angraecum* resulted from gradual species accumulation through time rather than rapid radiation.

## Supporting Information

S1 Fig **K-means cascade plot** showing the group attributed to (A) flower size and (B) spur length for each partition. Partitions with four (4) groups were selected for analyses for both characters.
(TIF)

S2 Fig Phylogenetic relationships of Angraecinae, 50% Bayesian majority-rule consensus tree from the nuclear ribosomal sequences (ITS2). Values above branches or at nodes represent posterior probability (PP) and bootstrap percentage (BP) support. Dashes represent branches that collapsed in the maximum parsimony strict consensus tree; taxa in bold are *Angraecum sensu* Garay. Abbreviations in brackets denote sections *sensu* Garay: Aca = *Acaulia*, Ang = *Angraecum*, Arc = *Arachnangraecum*, Bor = *Boryangraecum*, Fil = *Filangis*, Hum = *Humblotiangraecum*, Pct = *Pectinaria*, Per = *Perrierangraecum*, Psj = *Pseudojumellea*.
(TIF)

S3 Fig Phylogenetic relationships within subtribe Angraecinae, 50% Bayesian majority-rule consensus tree from plastid (*mat*K, *rps*16 and *trn*L) and morphological data. Values above branches or at nodes represent posterior probability (PP) and bootstrap percentage (BP) support. Dashes represent branches that collapsed in the maximum parsimony strict consensus tree; taxa in bold are *Angraecum sensu* Garay. Abbreviations in brackets denote sections *sensu* Garay: Aca = *Acaulia*, Agd = *Angraecoides*, Ang = *Angraecum*, Arc = *Arachnangraecum*, Bor = *Boryangraecum*, Chl = *Chlorangraecum*, Fil = *Filangis*, Gom = *Gomphocentrum*, Had = *Hadrangis*, Hum = *Humblotiangraecum*, Lem = *Lemurangis*, Lep = *Lepervenchea*, Pct = *Pectinaria*, Per = *Perrierangraecum*, Psj = *Pseudojumellea*.
(TIF)

S4 Fig Maximum credibility tree of the calibrated relaxed molecular clock analysis of Angraecinae inferred from combined plastid *mat*K, *rps*16 and *trn*L sequences. Posterior probabilities are displayed above branches in italics; node ages are indicated in bold, with blue bars representing the 95% highest height probability densities (HPD) of the node. Gray dashed-line indicates the Pliocene events. Abbreviations: A, *Angraecum*; At, *Aeranthes*; J, *Jumellea*.
(TIF)

S5 Fig Configuration shifts from the 95% credible set sampled by BAMM for the evolution of spur length across a phylogenetic tree of Angraecinae with evolutionary rates through time. The intensity of colors on branches reflects the instantaneous rate of phenotypic evolution (cool colors = slow, warm = fast). The red curve illustrates the mean speciation rate-through-time trajectory of Angraecinae in million years.
(TIF)

S6 Fig Ancestral state reconstructions of floral characters in Angraecinae implemented in ‘diversitree’; colors represent character states and pie charts represent the probability of ancestral states at node. Characters: (A) flower color, (B) flower size and (C) spur length.
(TIF)

S1 Table Voucher and sources of DNA data. Taxa, geographical origin, voucher information, and NCBI or BOLD accession numbers (*mat*K, *rps*16, *trn*L, ITS) for samples used in the study; sequences that are from different individuals are presented with voucher information in brackets. Blank denote unavailable sequences, “unvouchered” are sequences from living collections used in previous studies [1,3]. Unvouchered specimens have been identified by H.N. Andriananjamanantsoa and J. Andriatiana (TAN). Abbreviations: Af, Africa; Am, America; As, Asia; Com, Comoros; Md, Madagascar; Reu, Reunion; Sey, Seychelles.
(CSV)

S2 Table Morphological character description. Description of vegetative and floral morphological characters in Angraecinae.
(CSV)

S3 Table Morphological character matrix. Morphological character matrix used in the phylogenetic reconstruction.
(CSV)

S4 Table Characters used in BAMM analyses. Continuous characters used for the phenotypic/evolution model, K-means partitioning and fractioning used for the speciation/extinction model implemented in BAMM, values for flower size and spur length were log-transformed and fraction represents the percentage of samples used in the analyses for each clade (from 0 to 1).
(CSV)

S5 Table Ages of crown nodes of relevant clades presented in the phylogeny of Angraecinae inferred from BEAST. Comparison between the mean time to most recent common ancestor (MRCA) estimates and the node height highest posterior density (HPD) intervals at 95%.
(CSV)

S6 Table Comparaison of diversificaiton model used for the BiSSE and MuSSE analyses. Arrow represents transition allowed between states: ↔, reversible, →, irreversible. Parameters: lambda, speciation rate; mu, extinction rate; q, character transition rate. Character and character states: color of flower (0, green; 1, white); position of labellum (0, uppermost; 1, lowermost); size of flower (1, small; 2, medium; 3, large; 4, very large); spur length (1, short; 2, medium; 3, long; 4, very long). Abbreviations: AIC, Akaike information criterion; M, model tested; lnLik, Log likelihood.
(CSV)
