# Supplementary figures and images for: Diversification of Angraecum (Orchidaceae, Vandeae) in Madagascar: Revised Phylogeny Reveals Species Accumulation through Time Rather than Rapid Radiation
Source: PLoS One. 2016 Sep 26;11(9):e0163194. doi: 10.1371/journal.pone.0163194 (PMC5036805; doi:10.1371/journal.pone.0163194)

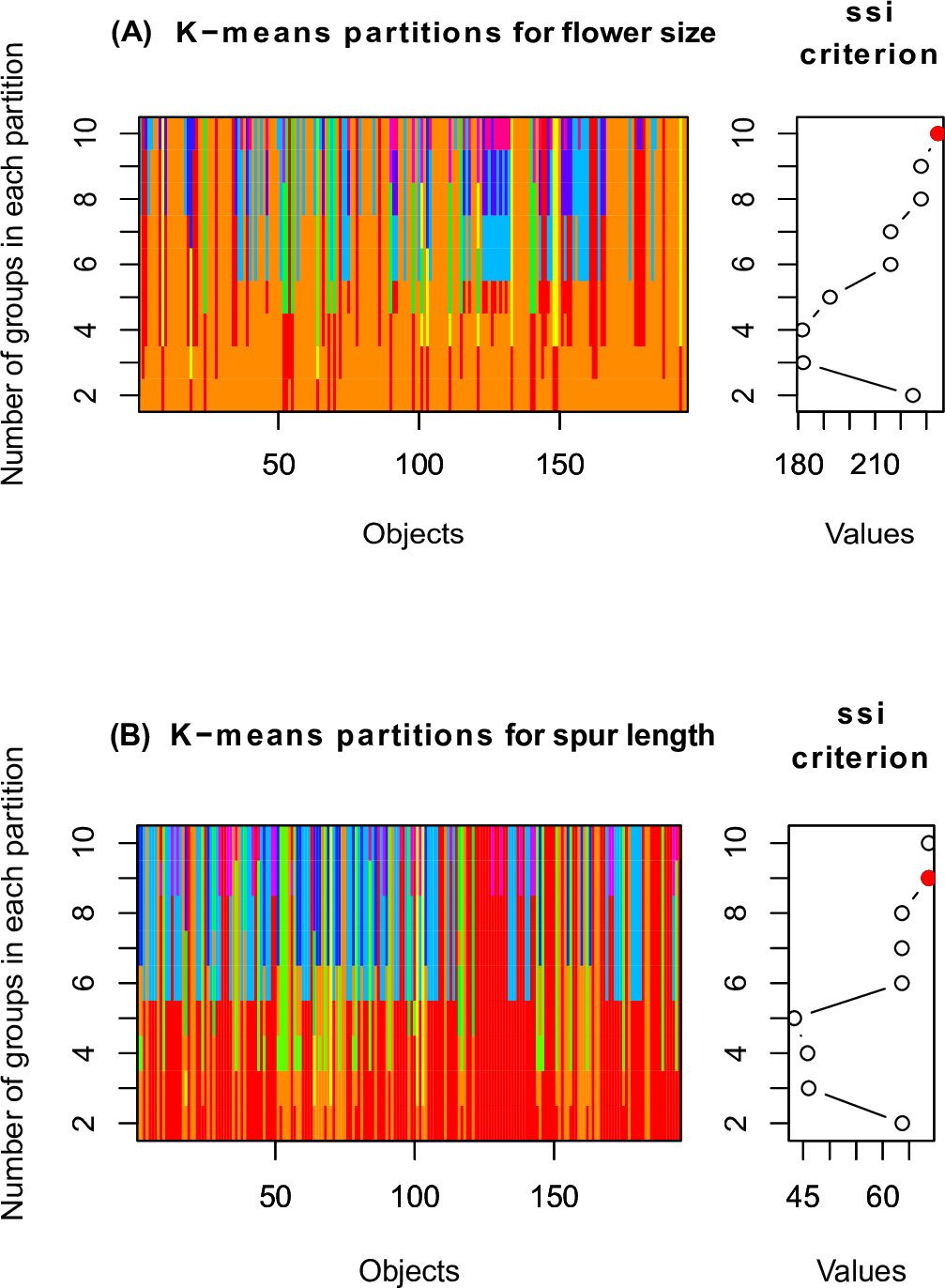

Supplement: S1 Fig — K-means cascade plot showing the group attributed to (A) flower size and (B) spur length for each partition. Partitions with four (4) groups were selected for analyses for both characters. (TIF) [file pone.0163194.s001.tif]

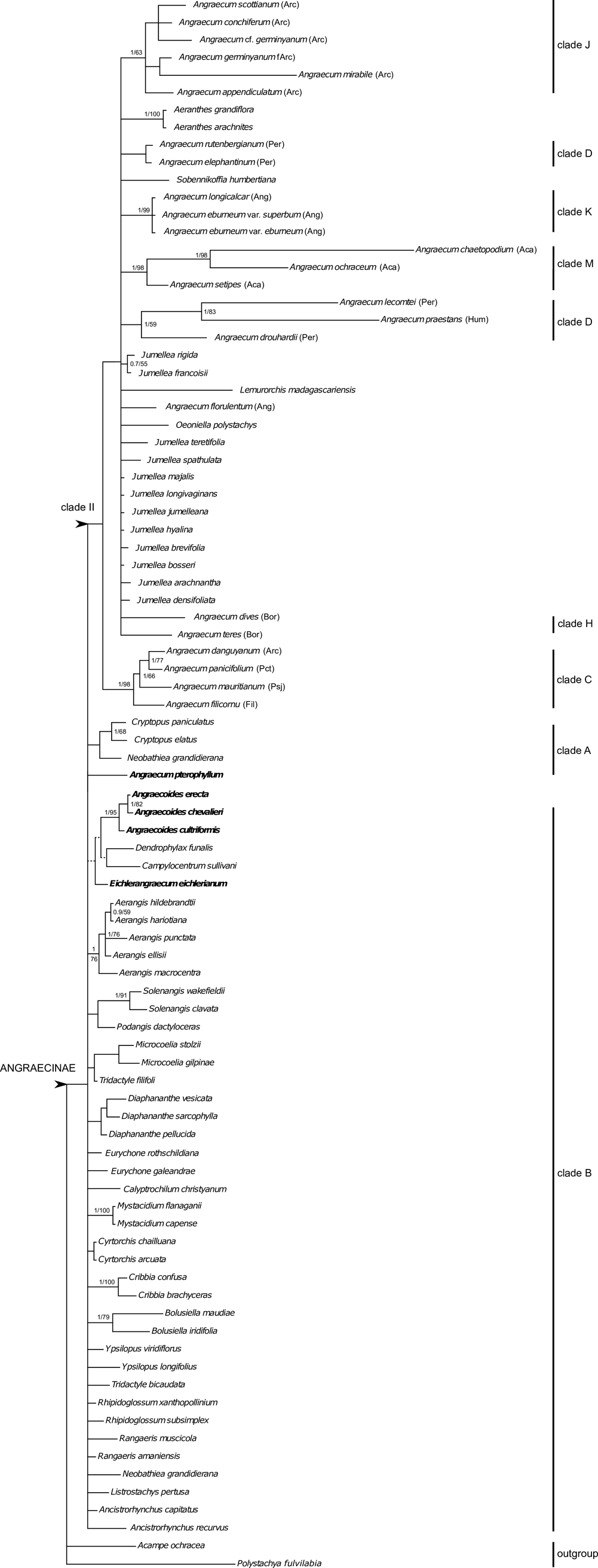

Supplement: S2 Fig — Values above branches or at nodes represent posterior probability (PP) and bootstrap percentage (BP) support. Dashes represent branches that collapsed in the maximum parsimony strict consensus tree; taxa in bold are Angraecum sensu Garay. Abbreviations in brackets denote sections sensu Garay: Aca = Acaulia, Ang = Angraecum, Arc = Arachnangraecum, Bor = Boryangraecum, Fil = Filangis, Hum = Humblotiangraecum, Pct = Pectinaria, Per = Perrierangraecum, Psj = Pseudojumellea. (TIF) [file pone.0163194.s002.tif]

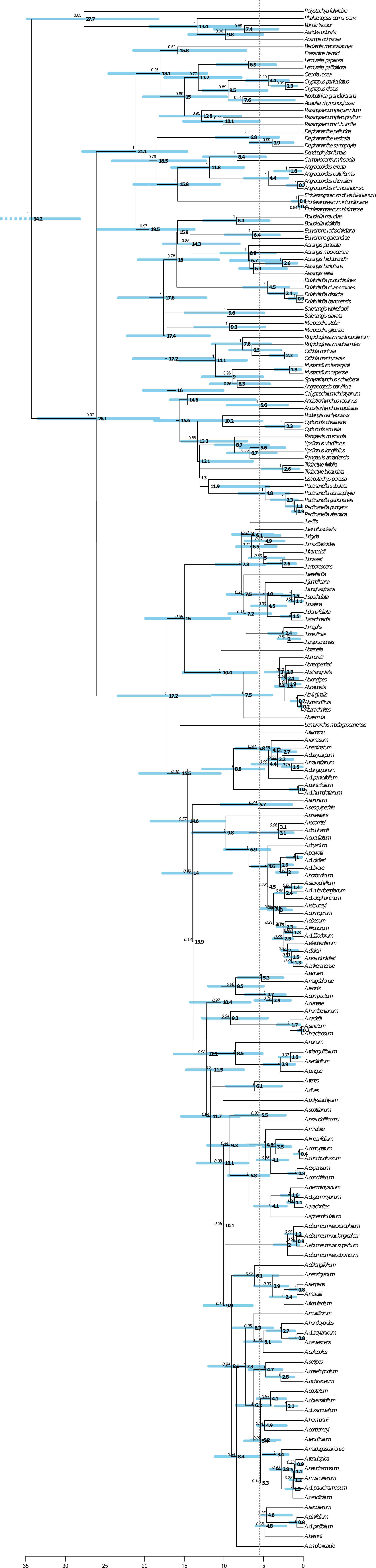

Supplement: S4 Fig — Posterior probabilities are displayed above branches in italics; node ages are indicated in bold, with blue bars representing the 95% highest height probability densities (HPD) of the node. Gray dashed-line indicates the Pliocene events. Abbreviations: A, Angraecum; At, Aeranthes; J, Jumellea. (TIF) [file pone.0163194.s004.tif]

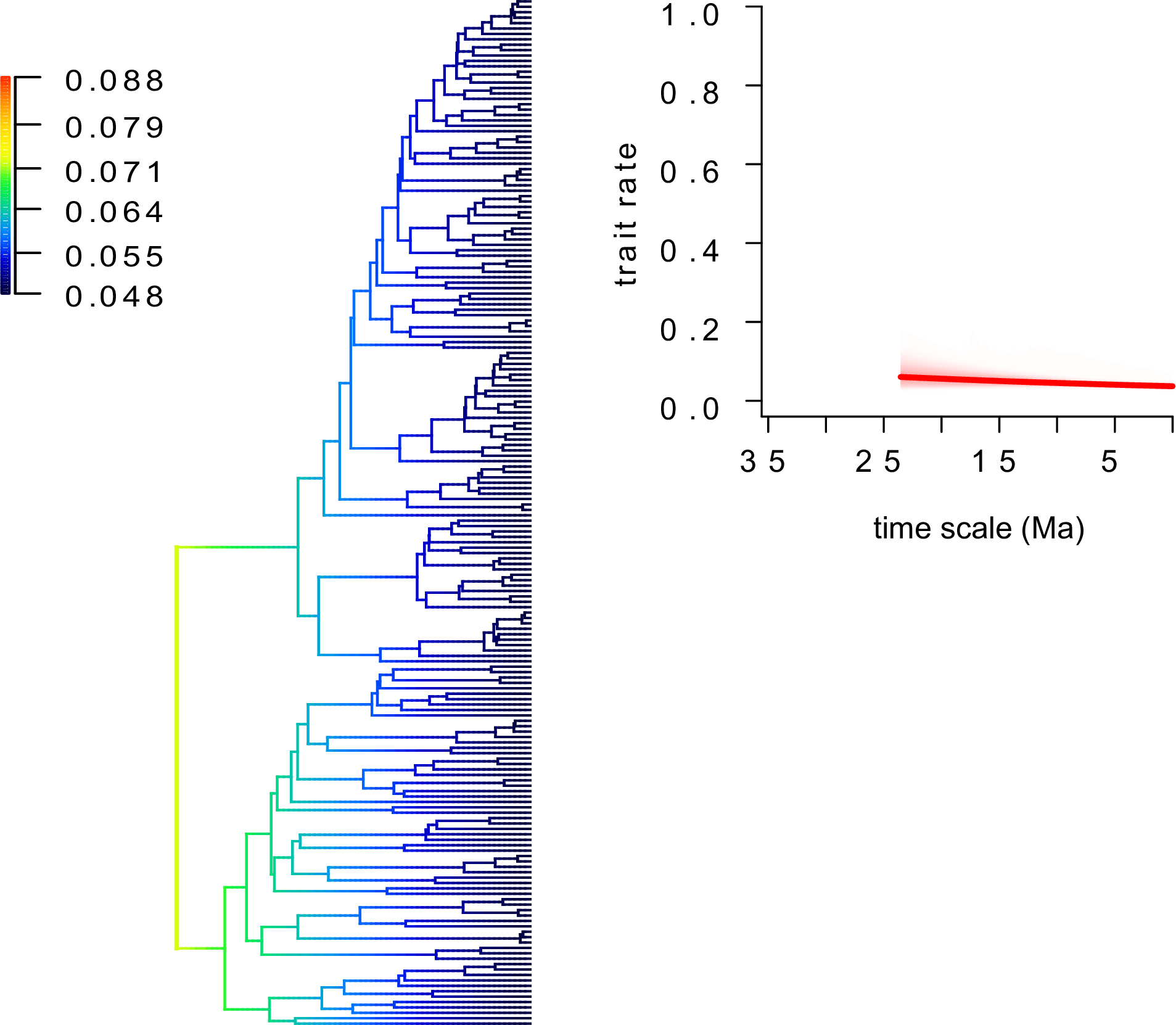

Supplement: S5 Fig — The intensity of colors on branches reflects the instantaneous rate of phenotypic evolution (cool colors = slow, warm = fast). The red curve illustrates the mean speciation rate-through-time trajectory of Angraecinae in million years. (TIF) [file pone.0163194.s005.tif]

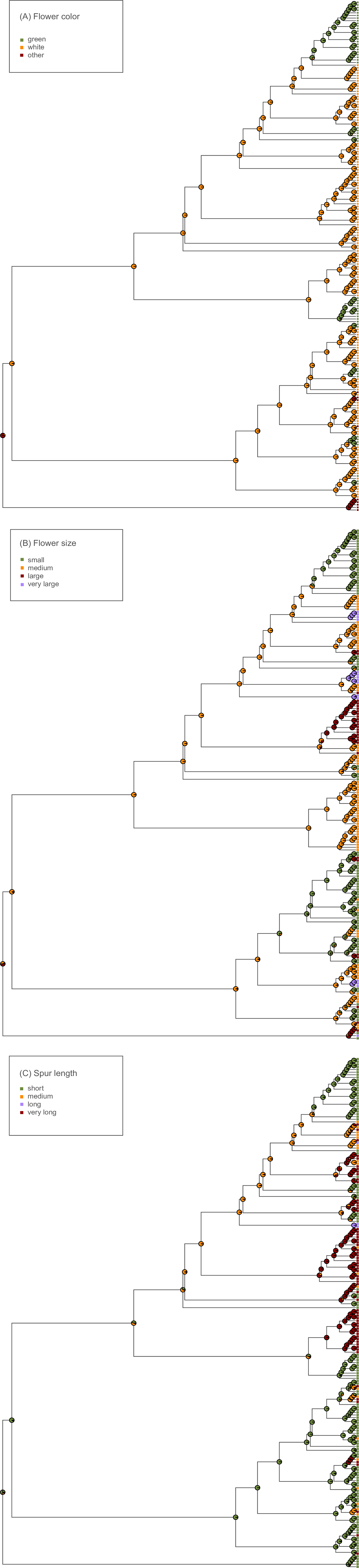

Supplement: S6 Fig — Characters: (A) flower color, (B) flower size and (C) spur length. (TIF) [file pone.0163194.s006.tif]
